# Supplementary material for: Blood transfusion and the risk for infections in kidney transplant patients
Source: PLoS One. 2021 Nov 12;16(11):e0259270. doi: 10.1371/journal.pone.0259270 (PMC8589196; doi:10.1371/journal.pone.0259270)
Supplement: S1 File — (DOCX) [file pone.0259270.s001.docx]

## APPENDIX – SUPPLEMENTARY MATERIAL

File S1: Usual immunosuppression protocol for kidney transplant at TOH

Kidney transplant recipients at TOH receive induction therapy with either thymoglobulin or basiliximab based on immunological risk, along will methylprednisone. When thymoglobulin is given, this is usually started in the immediate post-operative period. Those with pre-transplant cPRA ≥ 20% and those with early delayed graft function will receive thymoglobulin. ECD and DCD kidneys are considered at high risk for delayed graft function. All patients will also receive calcineurin inhibitor (nearly always tacrolimus), mycophenolate mofetil and prednisone as maintenance immunosuppression. The target trough for tacrolimus varies based on the period post-transplant, but once the 6-month mark is reached, typically the target trough is 4-6 ng/mL. The standard dose for mycophenolate mofetil is 1g twice a day. Oral prednisone is started early post-transplant and dose is tapered to reach 5mg daily around 12 weeks, and patients are typically kept on 5mg prednisone long-term.
