# Supplementary material for: Blood transfusion and the risk for infections in kidney transplant patients
Source: PLoS One. 2021 Nov 12;16(11):e0259270. doi: 10.1371/journal.pone.0259270 (PMC8589196; doi:10.1371/journal.pone.0259270)
Supplement: S1 Table — (DOCX) [file pone.0259270.s002.docx]

Table S1: RECORD guidelines checklist

|  | Item No. | Recommendation | Reported |
| --- | --- | --- | --- |
| Title and abstract | 1 | 1.1: The type of data used should be specified in the title or abstract. When possible, the name of the databases used should be included. | Title |
|  |  | 1.2: If applicable, the geographic region and time frame within which the study took place should be reported in the title or abstract. | Abstract |
|  |  | 1.3: If linkage between databases was conducted for the study, this should be clearly stated in the title or abstract. | Abstract |
| **Introduction** | | | |
| Background/  rationale | 2 | Explain the scientific background and rationale for the investigation being reported | Background |
| Objectives | 3 | State specific objectives, including any prespecified hypotheses | Background |
| **Methods** | | | |
| Study design | 4 | Present key elements of study design early in the paper | Methods – setting, participants and design |
| Setting | 5 | Describe the setting, locations and relevant dates, including periods of recruitment, exposure, follow-up and data collection | Methods – setting, participants and design |
| Participants | 6 | 6.1: The methods of study population selection (such as codes or algorithms used to identify subjects) should be listed in detail. If this is not possible, an explanation should be provided. | N/A |
|  |  | 6.2: Any validation studies of the codes or algorithms used to select the population should be referenced. If validation was conducted for this study and not published elsewhere, detailed methods and results should be provided. | N/A |
|  |  | 6.3: If the study involved linkage of databases, consider use of a flow diagram or other graphical display to demonstrate the data linkage process, including the number of individuals with linked data at each stage. | N/A |
| Variables | 7 | A complete list of codes and algorithms used to classify exposures, outcomes, confounders, and effect modifiers should be provided. If these cannot be reported, an explanation should be provided. | Methods, supplemental table S2 |
| Data sources/  Measurement | 8 | For each variable of interest, give sources of data and details of methods of assessment (measurement). Describe comparability of assessment methods if there is more than one group | Methods – outcomes; supplemental table S2 and S3 |
| Bias | 9 | Describe any efforts to address potential sources of bias | Methods – sensitivity analyses |
| Study size | 10 | Explain how the study size was arrived at | N/A |
| Quantitative variables | 11 | Explain how quantitative variables were handled in the analyses. If applicable, describe which groupings were chosen and why | Methods – statistical analysis |
| Statistical methods | 12 | (a) Describe all statistical methods, including those used to control for confounding. (b) Describe any methods used to examine subgroups and interactions. (c) Explain how missing data were addressed. (d) If applicable, explain how loss to follow-up was addressed. (e) Describe any sensitivity analyses. | Methods – statistical analysis, sensitivity analyses |
| Data access and cleaning | 12 | 12.1: Authors should describe the extent to which the investigators had access to the database population used to create the study population. | Methods – data sources |
|  |  | 12.2: Authors should provide information on the data cleaning methods used in the study. |  |
| Linkage | 12 | 12.3: State whether the study included person- level, institutional-level, or other data linkage across two or more databases. The methods of linkage and methods of linkage quality evaluation should be provided. | Methods – data sources |
| **Results** | | | |
| Participants | 13 | Describe in detail the selection of the persons included in the study (i.e., study population selection), including filtering based on data quality, data availability, and linkage. The selection of included persons can be described in the text and/or by means of the study flow diagram. | Methods – setting, participants, design; data sources |
| Descriptive data | 14 | (a) Give characteristics of study participants (e.g. demographic, clinical, social) and information on exposures and potential confounders | Results – patient characteristics, table 1 |
|  |  | (b) Indicate number of participants with missing data for each variable of interest | Methods – data sources |
|  |  | (c) Summarise follow-up time (e.g. average and total amount) | Results |
| Outcome data | 15 | Report numbers of outcome events or summary measures over time | Results – figure 2 and table 3 |
| Main results | 16 | (a) Give unadjusted estimates and, if applicable, confounder-adjusted estimates and their precision (e.g. 95% confidence interval). Make clear which confounders were adjusted for and why they were included | Results – association of RBCT with infections, table 4 |
|  |  | (b) Report category boundaries when continuous variables were categorized | Results - patient and transfusion characteristics |
|  |  | (c) If relevant, consider translating estimates of relative risk into absolute risk for a meaningful time period | N/A |
| Other analyses | 17 | Report other analyses done – e.g. analyses of subgroups and interactions, and sensitivity analyses | Results – sensitivity analyses |
| **Discussion** | | | |
| Key results | 18 | Summarise key results with reference to study objectives | Discussion |
| Limitations | 19 | Discuss the implications of using data that were not created or collected to answer the specific research question(s). Include discussion of misclassification bias, unmeasured confounding, missing data, and changing eligibility over time, as they pertain to the study being reported. | Discussion |
| Interpretation | 20 | Give a cautious overall interpretation of results considering objectives, limitations, multiplicity of analyses, results from similar studies, and other relevant evidence | Discussion |
| Gerneralisability | 21 | Discuss the gerneralisability (external validity) of the study results | Discussion |
| **Other Information** | | | |
| Funding | 22 | Give the source of funding and the role of the funders for the present study and, if applicable, for the original study on which the present article is based | End of manuscript |
| Accessibility of protocol, raw data and programming code | 22 | Authors should provide information on how to access any supplemental information such as the study protocol, raw data, or programming code | End of manuscript |

N/A not applicable
