# Supplementary material for: Blood transfusion and the risk for infections in kidney transplant patients
Source: PLoS One. 2021 Nov 12;16(11):e0259270. doi: 10.1371/journal.pone.0259270 (PMC8589196; doi:10.1371/journal.pone.0259270)
Supplement: S2 Table — (DOCX) [file pone.0259270.s003.docx]

Table S2: ICD10 codes used for identifying infections

|  | **ICD10 code** | **Description** |
| --- | --- | --- |
| Pneumonia | J11 | Influenza, virus not identified |
|  | J12 | Viral pneumonia, not elsewhere classified |
|  | J13 | Pneumonia due to Streptococcus pneumoniae |
|  | J14 | Pneumonia due to Haemophilus influenzae |
|  | J15 | Bacterial pneumonia, not elsewhere classified |
|  | J16 | Pneumonia due to other infectious organisms, not elsewhere classified |
|  | J17 | Pneumonia in diseases classified elsewhere |
|  | J18 | Pneumonia, organism unspecified |
| Acute pyelonephritis | N10 | Acute tubule-interstitial nephritis (acute infectious interstitial nephritis, acute pyelitis, acute pyelonephritis) |
| Urinary tract infection | N39.0 | Urinary tract infection, site not specified |
| Sepsis | A41.0 | Sepsis due to Staphylococcus aureus |
|  | A41.1 | Sepsis due to other specified staphylococcus |
|  | A41.2 | Sepsis due to unspecified staphylococcus |
|  | A41.3 | Sepsis due to Haemophilus influenzae |
|  | A41.4 | Sepsis due to anaerobes |
|  | A41.5 | Sepsis due to other Gram-negative organisms |
|  | A41.8 | Other specified sepsis |
|  | A41.9 | Sepsis, unspecified |
|  | R57.2 | Septic shock |
|  | R65.1 | Systemic Inflammatory Response Syndrome of infectious origin with organ failure |
