# Supplementary material for: Blood transfusion and the risk for infections in kidney transplant patients
Source: PLoS One. 2021 Nov 12;16(11):e0259270. doi: 10.1371/journal.pone.0259270 (PMC8589196; doi:10.1371/journal.pone.0259270)
Supplement: S3 Table — (DOCX) [file pone.0259270.s004.docx]

Table S3: Baseline characteristics ascertained for the study

| **Characteristic** | **Data source** |
| --- | --- |
| Age at transplant date | OHDW |
| Sex | OHDW |
| Race (caucasian, black, asian, middle-eastern, other) | Chart |
| Cause of ESKD (GN, diabetes, PCKD, CAKUT, other) | Chart |
| Type of kidney transplant (living or deceased donor) | TOH renal transplant database |
| Number of the current kidney transplant | Chart |
| Recipient re-transplanted during study period | Chart |
| Diabetes (ICD code in the 5 years preceding transplant) | OHDW  ICD9: 250  ICD10: E10, E11, E12, E13, E14 |
| Cardiovascular disease (ICD10 code for CAD, CHF, AFib or ischemic stroke in the 5 years preceding transplant) | OHDW  CAD   - ICD9: 410, 411, 412, 413, 414, 4292, 4296, 4297 - ICD10: I20, I21, I22, I23, I24, I25, Z955, Z958, Z959, R931, T822   CHF   - ICD9: 425, 428, 514, 5184 - ICD10: I500, I501, I509, I255, J81   AFib   - ICD9: 4273 - ICD10: I48   Stroke   - ICD9: 434, 436 - ICD10: H341, I630, I631, I632, I633, I634, I635, I638, I639, I64 |
| Most recent PRA prior to transplant | Chart |
| Type of induction therapy used (T-cell depleting or non-T-cell depleting)^†^ | OHDW |
| Initial maintenance therapy prescribed (tacrolimus or other) | OHDW |

* GN glomerulonephritis; PCKD polycystic kidney disease; CAKUT congenital anomalies of the kidneys and urinary tracts; CAD coronary artery disease; CHF congestive heart failure; AFib atrial fibrillation; PRA panel reactive antibodies; OHDW ottawa hospital data warehouse

^†^ At our institution, kidney transplant recipients typically receive t-cell depleting induction therapy in the immediate post-operative when the immunological risk is considered high (pre-transplant PRA >20% or onset of DGF post-transplant)
