# Supplementary material for: Blood transfusion and the risk for infections in kidney transplant patients
Source: PLoS One. 2021 Nov 12;16(11):e0259270. doi: 10.1371/journal.pone.0259270 (PMC8589196; doi:10.1371/journal.pone.0259270)
Supplement: S4 Table — (DOCX) [file pone.0259270.s005.docx]

Table S4: Association of RBCT with outcomes for different time-lags between exposure and occurrence of outcome (HR [95% CI])

|  | Original analysis | 3-day lag | 7-day lag | 10-day lag | 14-day lag |
| --- | --- | --- | --- | --- | --- |
| Bacterial infection | | | | | |
| **RBC category**  None  1  2  3-5  >5 | Reference  1.35 (0.95 to 1.91)  1.29 (0.92 to 1.82)  2.63 (1.94 to 3.56)  3.38 (2.30 to 4.95) | Reference  1.29 (0.91 to 1.84)  1.33 (0.96 to 1.86)  2.44 (1.79 to 3.32)  3.20 (2.18 to 4.71) | Reference  1.23 (0.86 to 1.75)  1.17 (0.83 to 1.64)  2.18 (1.60 to 2.98)  2.32 (1.52 to 3.54) | Reference  1.03 (0.71 to 1.50)  1.15 (0.82 to 1.62)  2.05 (1.50 to 2.81)  1.97 (1.27 to 3.07) | Reference  1.00 (0.69 to 1.46)  1.04 (0.73 to 1.47)  1.90 (1.39 to 2.61)  1.73 (1.10 to 2.73) |
| Viral infection | | | | | |
| **RBC category**  None  1  2  3-5  >5 | Reference  1.41 (0.80 to 2.47)  0.86 (0.40 to 1.82)  1.96 (1.03 to 3.74)  1.06 (0.25 to 4.52) | Reference  1.33 (0.76 to 2.32)  0.60 (0.26 to 1.41)  1.68 (0.86 to 3.26)  1.00 (0.24 to 4.27) | Reference  1.33 (0.76 to 2.32)  0.80 (0.38 to 1.70)  1.37 (0.67 to 2.82)  1.00 (0.24 to 4.28) | Reference 1.38 (0.80 to 2.39)  0.59 (0.25 to 1.37)  1.34 (0.65 to 2.75)  0.98 (0.23 to 4.18) | Reference  1.38 (0.80 to 2.39)  0.59 (0.25 to 1.37)  1.34 (0.65 to 2.75)  0.98 (0.23 to 4.18) |

RBCT, red blood cell transfusion
