# Supplementary material for: Blood transfusion and the risk for infections in kidney transplant patients
Source: PLoS One. 2021 Nov 12;16(11):e0259270. doi: 10.1371/journal.pone.0259270 (PMC8589196; doi:10.1371/journal.pone.0259270)
Supplement: S5 Table — (DOCX) [file pone.0259270.s006.docx]

Table S5: Risks for any infection (bacterial or viral) by cumulative RBCT exposure

The cohort for this analysis is limited to transplant recipients March 14, 2014 onwards since this is when CMV viremia data was available in our database.

|  | # RBC units received | # events (%) | Time-varying crude HR (95% CI) | Time-varying adjusted HR (95% CI)* |
| --- | --- | --- | --- | --- |
| March 14, 2014 onwards | None  1  2  3-5  >5 | 104 (34.0)  25 (53.2)  9 (22.0)  25 (64.1)  14 (73.7) | Reference  1.72 (1.11 to 2.67)  0.69 (0.35 to 1.36)  2.70 (1.61 to 4.57)  4.59 (1.69 to 12.51) | Reference  1.34 (0.85 to 2.13)  0.57 (0.29 to 1.15)  2.39 (1.38 to 4.12)  4.27 (1.53 to 11.89) |

* Adjusted for age, sex, transplant type, cause of ESKD, PRA, presence of CVD, receipt of t-cell depleting induction and type of maintenance therapy.
