# Supplementary material for: Blood transfusion and the risk for infections in kidney transplant patients
Source: PLoS One. 2021 Nov 12;16(11):e0259270. doi: 10.1371/journal.pone.0259270 (PMC8589196; doi:10.1371/journal.pone.0259270)
Supplement: S6 Table — (DOCX) [file pone.0259270.s007.docx]

Table S6: Time-varying, adjusted hazard ratios (95% CI) for infections, stratified by DGF status of kidney transplant

|  | # RBC units received | Original analysis | DGF Yes | DGF No |
| --- | --- | --- | --- | --- |
| Bacterial infection | None  1  2  3-5  >5 | Reference  1.35 (0.95 to 1.91)  1.29 (0.92 to 1.82)  2.63 (1.94 to 3.56)  3.38 (2.30 to 4.95) | Reference  1.82 (1.00 to 3.29)  1.16 (0.67 to 2.02)  2.59 (1.59 to 4.21)  2.97 (1.64 to 5.35) | Reference  0.97 (0.61 to 1.55)  1.28 (0.81 to 2.02)  2.02 (1.30 to 3.14)  3.98 (2.33 to 6.82) |
| Viral infection | None  1  2  3-5  >5 | Reference  1.56 (0.88 to 2.75)  0.94 (0.44 to 2.00)  1.96 (1.00 to 3.83)  1.13 (0.27 to 4.85) | Reference  2.94 (1.03 to 8.37)  1.38 (0.42 to 4.59)  3.79 (1.18 to 12.13  0.77 (0.09 to 6.87) | Reference  1.33 (0.63 to 2.81)  1.13 (0.34 to 3.77)  1.52 (0.53 to 4.35)  2.85 (0.37 to 21.77) |
