# Supplementary material for: Blood transfusion and the risk for infections in kidney transplant patients
Source: PLoS One. 2021 Nov 12;16(11):e0259270. doi: 10.1371/journal.pone.0259270 (PMC8589196; doi:10.1371/journal.pone.0259270)
Supplement: S7 Table — (DOCX) [file pone.0259270.s008.docx]

Table S7: Association of RBCT with outcomes accounting for RBCT occurring on day of transplant surgery

In our study, we were unable to know if a RBCT given on the day of kidney transplant was given pre- or post-transplant. This is because we did not have the exact time of transplant and a deceased donor transplant may occur at any time during the day or night. For this reason, we decided to classify RBCT exposure from day 1 post-transplant onwards. This, however, carries the risk of misclassifying an individual as unexposed if they got transfused post-transplant on the day of transplant and never again afterwards. Also, it could affect the time-varying exposure analysis if an individual was transfused post-transplant on the day of transplant and the next RBCT was only a few months later.

There were 104 study observations who received a RBCT on the day of transplant surgery; 42 of these only received RBCT on the day of transplant and never again, and 62 of these received subsequent transfusion(s) from day 1 onwards. We therefore randomly assigned a first RBCT as occurring at time 0.01 days post-transplant to 50% of these 104 observations (considering that probably about half of those transfused on the day of surgery actually received the RBCT post-transplant and not pre-transplant). The 50% is based on an educated guess since at our center, not so rarely are patients given a transfusion pre-operatively, as requested by either anesthesia or the surgeons, as a “top-up” prior to surgery even if there is no strong indication. We then carried out the same time-varying analysis with the new RBCT exposure on day 0 for the 52 randomly chosen observations. We found overall similar results (see table below).

|  | # RBC units received | Time-varying adjusted HR (95% CI); original analysis | Time-varying adjusted HR (95% CI); re-assigned day 0 RBC exposure |
| --- | --- | --- | --- |
| Bacterial infection | None  1  2  3-5  >5 | Reference  1.35 (0.95 to 1.91)  1.29 (0.92 to 1.82)  2.63 (1.94 to 3.56)  3.38 (2.30 to 4.95) | Reference  1.16 (0.83 to 1.63)  1.42 (1.02 to 1.98)  2.31 (1.71 to 3.11)  3.40 (2.33 to 4.96) |
| Viral infection | None  1  2  3-5  >5 | Reference  1.41 (0.80 to 2.47)  0.86 (0.40 to 1.82)  1.96 (1.03 to 3.74)  1.06 (0.25 to 4.52) | Reference  1.37 (0.78 to 2.42)  0.78 (0.37 to 1.66)  1.84 (0.97 to 3.50)  1.02 (0.24 to 4.36) |

RBC, red blood cell
