# Supplementary material for: Blood transfusion and the risk for infections in kidney transplant patients
Source: PLoS One. 2021 Nov 12;16(11):e0259270. doi: 10.1371/journal.pone.0259270 (PMC8589196; doi:10.1371/journal.pone.0259270)
Supplement: S8 Table — (DOCX) [file pone.0259270.s009.docx]

### Table S8: Time-varying, adjusted hazard ratios (95% CI) for outcomes, controlling for year of transplantation

Since our study period spans 17 years, one can expect there to be changes in surgical practice, quality of care for kidney transplant recipients, anemia management as well as changes in the methods of diagnosing rejection. To account for these changes over time and whether or not it would change our results, re-analysis was done for all outcomes after adding a variable “txyear” in the model which represents the calendar year when the kidney transplant occurred for each observation. As can be seen below, for most outcomes the HRs attenuated slightly, or they remained nearly the same. Therefore, the overall study conclusions are not affected.

|  | # RBC units received | Original analysis | Controlling for year of transplant |
| --- | --- | --- | --- |
| Bacterial infection | None  1  2  3-5  >5 | Reference  1.35 (0.95 to 1.91)  1.29 (0.92 to 1.82)  2.63 (1.94 to 3.56)  3.38 (2.30 to 4.95) | Reference  1.35 (0.95 to 1.91)  1.30 (0.92 to 1.83)  2.64 (1.95 to 3.59)  3.40 (2.31 to 4.98) |
| Viral infection | None  1  2  3-5  >5 | Reference  1.41 (0.80 to 2.47)  0.86 (0.40 to 1.82)  1.96 (1.03 to 3.74)  1.06 (0.25 to 4.52) | Reference  1.40 (0.80 to 2.45)  0.87 (0.41 to 1.85)  1.93 (1.01 to 3.66)  1.12 (0.26 to 4.81) |

RBC, red blood cell
