# Supplementary material for: Blood transfusion and the risk for infections in kidney transplant patients
Source: PLoS One. 2021 Nov 12;16(11):e0259270. doi: 10.1371/journal.pone.0259270 (PMC8589196; doi:10.1371/journal.pone.0259270)
Supplement: S9 Table — (DOCX) [file pone.0259270.s010.docx]

Table S9: Transfusion frequency among those with infection, whether the infection occurred after a rejection or not

|  | Never transfused (N=790) | Transfused during study (N=468) | p-value |
| --- | --- | --- | --- |
| Infection after rejection; N (%) | 11 (1.4%) | 40 (8.6%) | <0.0001 |
| Infection not after rejection, i.e. no rejection or infection occurred prior to rejection; N (%) | 194 (24.6%) | 211 (45.1%) | <0.0001 |
