# Supplementary material for: Blood transfusion and the risk for infections in kidney transplant patients
Source: PLoS One. 2021 Nov 12;16(11):e0259270. doi: 10.1371/journal.pone.0259270 (PMC8589196; doi:10.1371/journal.pone.0259270)
Supplement: S10 Table — (DOCX) [file pone.0259270.s011.docx]

Table S10: Time-varying, adjusted hazard ratios (95% CI) for outcomes, when restricting follow-up to the first 90-days post-transplant

Most transfusions occurred during the first week after transplant. However, most infections occurred many months to over a year after transplant (median days to bacterial infection 409 and to viral infection 154). We therefore chose to examine the infectious risks associated with RBCT by restricting the follow-up of all study participants to the first 90-days post-transplant. By doing so, this focuses on the period when the time-frame between RBCT and infection would be shorter, and the possible immunomodulatory effect from RBCT more likely to impact occurrence of infection. As can be seen below, we found similar results to our original analysis.

|  | # RBC units received | Original analysis | 90-day follow-up |
| --- | --- | --- | --- |
| Bacterial infection | None  1  2  3-5  >5 | Reference  1.35 (0.95 to 1.91)  1.29 (0.92 to 1.82)  2.63 (1.94 to 3.56)  3.38 (2.30 to 4.95) | Reference  1.81 (1.01 to 3.26)  1.42 (0.76 to 2.67)  4.10 (2.40 to 7.03)  6.58 (3.24 to 13.36) |
| Viral infection (BK or CMV) | None  1  2  3-5  >5 | Reference  1.41 (0.80 to 2.47)  0.86 (0.40 to 1.82)  1.96 (1.03 to 3.74)  1.06 (0.25 to 4.52) | Reference  2.57 (0.92 to 7.19)  2.29 (0.71 to 7.43)  1.92 (0.40 to 9.13)  N/A |
