# Supplementary material for: Blood transfusion and the risk for infections in kidney transplant patients
Source: PLoS One. 2021 Nov 12;16(11):e0259270. doi: 10.1371/journal.pone.0259270 (PMC8589196; doi:10.1371/journal.pone.0259270)
Supplement: S1 Fig — (DOCX) [file pone.0259270.s012.docx]

Figure S1: Mean hemoglobin level pre-transfusion by year of transfusion

Caption: The mean hemoglobin value of all blood transfusions issued per year decreased over time. The hemoglobin value represents the most recent hemoglobin in the 1-48 hours preceding the issuing of the transfusion, therefore likely representing the hemoglobin value which prompted transfusion. This decrease over time likely reflects changes in overall transfusion practice as restrictive transfusion strategies have been more widely recommended and adopted over the years. All transfusions at our institution are given at the discretion of the treating physician and would therefore be thought to have considered transfusion guidelines and the patient’s clinical condition.
