# Supplementary material for: Blood transfusion and the risk for infections in kidney transplant patients
Source: PLoS One. 2021 Nov 12;16(11):e0259270. doi: 10.1371/journal.pone.0259270 (PMC8589196; doi:10.1371/journal.pone.0259270)
Supplement: Attachment — Submitted filename: Rebuttal document - Editor and Reviewer comments.docx [file pone.0259270.s013.docx]

 **The line numbers indicating where changes were made pertain to the “clean” version of the corrected manuscript**

Journal Requirements:

When submitting your revision, we need you to address these additional requirements.

1. Please ensure that your manuscript meets PLOS ONE's style requirements, including those for file naming. The PLOS ONE style templates can be found at 
https://journals.plos.org/plosone/s/file?id=wjVg/PLOSOne_formatting_sample_main_body.pdf and 
https://journals.plos.org/plosone/s/file?id=ba62/PLOSOne_formatting_sample_title_authors_affiliations.pdf

**This has been done in the corrected version**

2. Please include your full ethics statement in the ‘Methods’ section of your manuscript file. In your statement, please include the full name of the IRB or ethics committee who approved or waived your study, as well as whether or not you obtained informed written or verbal consent. If consent was waived for your study, please include this information in your statement as well.

**We have included the name of our research and ethics board on lines 129-130 of the corrected manuscript. We direct your attention to the statement mentioning informed consent was waived due to the retrospective nature of our study as per our institution’s requirements.**

3. Thank you for stating in the text of your manuscript "Due to the retrospective nature of our study and the use of deidentified data, informed consent was waived.". Please also add this information to your ethics statement in the online submission form.

**We shall do so on the online submission form**

4. Please include your actual numerical p-values in Table 4

**We have included the p-values as an extra column in Table 4**

5. We note that you have indicated that data from this study are available upon request. PLOS only allows data to be available upon request if there are legal or ethical restrictions on sharing data publicly. For more information on unacceptable data access restrictions, please see http://journals.plos.org/plosone/s/data-availability#loc-unacceptable-data-access-restrictions.

In your revised cover letter, please address the following prompts:

a) If there are ethical or legal restrictions on sharing a de-identified data set, please explain them in detail (e.g., data contain potentially sensitive information, data are owned by a third-party organization, etc.) and who has imposed them (e.g., an ethics committee). Please also provide contact information for a data access committee, ethics committee, or other institutional body to which data requests may be sent.

**Our apologies, the statement that data is available upon request is incorrect. Our ethics board does not allow private health information to be made publicly available, in accordance with our province’s Public Health Information Protection Act. Indeed, the data contain sensitive health information (date of birth, race, sex, date of transplant) which could make an individual identifiable.**

b) If there are no restrictions, please upload the minimal anonymized data set necessary to replicate your study findings as either Supporting Information files or to a stable, public repository and provide us with the relevant URLs, DOIs, or accession numbers. For a list of acceptable repositories, please see http://journals.plos.org/plosone/s/data-availability#loc-recommended-repositories.

**See above**

6. PLOS requires an ORCID iD for the corresponding author in Editorial Manager on papers submitted after December 6th, 2016. Please ensure that you have an ORCID iD and that it is validated in Editorial Manager. To do this, go to ‘Update my Information’ (in the upper left-hand corner of the main menu), and click on the Fetch/Validate link next to the ORCID field. This will take you to the ORCID site and allow you to create a new iD or authenticate a pre-existing iD in Editorial Manager. Please see the following video for instructions on linking an ORCID iD to your Editorial Manager account: <https://www.youtube.com/watch?v=_xcclfuvtxQ>

**Added as requested**

7.  Thank you for submitting the above manuscript to PLOS ONE. During our internal evaluation of the manuscript, we found significant text overlap between your submission and the following previously published work, of which you are an author.

- https://www.kireports.org/article/S2468-0249(21)00017-6/fulltext

We would like to make you aware that copying extracts from previous publications, especially outside the methods section, word-for-word is unacceptable. In addition, the reproduction of text and data from published reports has implications for the copyright that may apply to the publications.

Please revise the manuscript to rephrase the duplicated text, cite your sources, and provide details as to how the current manuscript advances on previous work. Please note that further consideration is dependent on the submission of a manuscript that addresses these concerns about the overlap in text with published work.

We will carefully review your manuscript upon resubmission, so please ensure that your revision is thorough

**We share your concerns about overlap and worked to ensure the present work is distinct and unique. Yes indeed, this study was part of an overarching project by our group examining the comprehensive risks associated with blood transfusions in kidney transplant patients. This was D.M-A’s Master’s thesis project. The first study (the one in KIReports which you mention above) was the first study of this project looking at the risks for adverse graft outcomes associated with transfusions. The current study (manuscript currently submitted to PLOSOne) focuses on the clinically common risk for infections. Therefore, the methods and cohort are highly similar; however the importance of the research question is distinct and as such we felt a separate manuscript was required to fully, appropriately and adequately address the question. We have revised the methods to reference the previous work and worked to differentiate from the previous studies as follows: i) a statement was added at the start of the methods (lines 114-118) referencing our previous work and explicitly stating the methods are similar to the previously published study, ii) a similar statement was added to the Results section with the again, the appropriate reference (lines 234-235); iii) key parts of the methods and results section were rephrased. Similarities remain in the data describing the baseline characteristics of the cohort, the study cohort creation and descriptors of transfusions received in the population with an appropriate reference added.**

**We thank you for bringing this important point to our attention. We hope that adding the statements as detailed above, citing the study and re-phrasing key parts of the manuscript will suffice.**

**Review Comments to the Author**

 **The line numbers indicating where changes were made pertain to the “clean” version of the corrected manuscript**

**Reviewer #1**: I wish to compliment the authors with regard to their manuscript.
As a whole I consider this to be a comprehensive paper.

However I am confused with regard to the presented analysis. If I understand the manuscript correctly the authors focused extensively upon the question if early infections in relation to transfusion could be two signs of the same underlying cause and therefor there might reflect some bias.
However I can't find a proper evaluation between the timing of the transfusion and the timing of the first endpoint. If I understand the analysis correctly then even ten years after transfusion there appears to be a higher incidence of bacterial infection.
I would like more information on the time relation between transfusion and the occurrence of the first end point. What is the median/ mean duration in weeks or days. One would expect for an immunomodulatory effect of a transfusion to occur and affect the chances of infection; too short an interval does not feel likely however too long an interval seems equally unlikely; one would assume that such an effect would disappear over time.
If this risk indeed persists over ten years as I comprehend from the manuscript I have two questions for the authors:
- If a higher rate of infection can be evaluated so long after transfusion then in the analysis some attention should be paid to transfusions prior to transplantation; as they might affect the observed incidences (assuming that those transfusions also affect the chance of infections for years).
- If a higher rate of infection can be evaluated so long after transfusion I would like to invite the authors to propose an explanation for this phenomenon

In examining figure 2 one could also propose that two years after transfusion the (annual) rate of infection between transfused and non-transfused patients actually appears to be similar and that the effect of transfusion on infection rate actually is a temporary effect which seems to disappear over time.
Taking this into account one could wonder if a ten year follow-up for the analyses is appropriate. Some reflection on the timing of achievement of the endpoint after transfusion is required.

**The issue of time of occurrence of infection relative to RBCT is a good point brought up by the reviewer and we agree that adding more information about this could be informative to the reader. We have added the median time from first transfusion to occurrence of bacterial and viral infection, among those who had infection occur after RBCT, in the Results section (lines 268-269 and 273-274). We unfortunately could not know the amount of RBCT given to our patients prior to transplant since most patients receive dialysis at peripheral dialysis centers and clinics where we would not be able to capture transfusion data.**

**Furthermore, because it is true that there is a significant time period between RBCT and infection in our study, we performed another additional analysis where we restricted the follow-up to the first 90-days after transplant, a period when the time between RBCT and infection would be shorter, thus making a potential immunomodulatory effect from RBCT more likely. We present the results of this extra analysis in the table below and have added this in our manuscript (Methods lines 225-227 and Results lines 318-319). We have also added this table as a supplementary appendix (Table S10 in the Supporting Information document). As you will see, the findings are similar to our original analysis.**

|  | # RBC units received | Original analysis | 90-day follow-up |
| --- | --- | --- | --- |
| Bacterial infection | None  1  2  3-5  >5 | Reference  1.35 (0.95 to 1.91)  1.29 (0.92 to 1.82)  2.63 (1.94 to 3.56)  3.38 (2.30 to 4.95) | Reference  1.81 (1.01 to 3.26)  1.42 (0.76 to 2.67)  4.10 (2.40 to 7.03)  6.58 (3.24 to 13.36) |
| Viral infection (BK or CMV) | None  1  2  3-5  >5 | Reference  1.41 (0.80 to 2.47)  0.86 (0.40 to 1.82)  1.96 (1.03 to 3.74)  1.06 (0.25 to 4.52) | Reference  2.57 (0.92 to 7.19)  2.29 (0.71 to 7.43)  1.92 (0.40 to 9.13)  N/A |

**It is true that most of the separation between those transfused and not transfused in terms of occurrence of infections happens in the first 1-2 years after transplant based on our Figure 2. We chose to examine our outcomes with as long follow-up as possible before having seen the results, which is why we feel we should keep our results presented as such. However, we have added a mention of the fact that most of the increased risk for infection seems to occur in the first year after transplant, which is probably because nearly all RBCT occurred in the first week after transplant and the early period is when transplant patients are at greatest risk for infections (Discussion lines 353-356).**

**Reviewer #2**: Thank you for asking me to review this submission from Massicotte-Azarniouch and colleagues exploring use of red blood cell transfusion after kidney transplantation and risk for bacterial/viral infection, finding a positive association with bacterial infections only. The manuscript is interesting, well written and deals with an important issue.

My comments to help better understand the submission and aid revisions are:

1. On page 8 the authors state 'DGF is considered high-immunological risk at our
196 institution and such patients receive thymoglobulin'. How are people at risk for DGF identified prior to transplant? ECD kidneys? DCD kidneys?

**Indeed, ECD and DCD kidneys are considered at risk for DGF. We have added this information as a supplemental file which summarizes our immunosuppression use in our kidney transplant recipients (see Supporting Information File S1).**

2. The statistical analyses are comprehensive. The authors mention they censored for death/graft loss but I wonder whether they should consider doing a competing risk analysis? Clearly a kidney transplant patient who dies or loses their kidney is no longer at risk of exposure to the outcome of interest.

**We believe the reviewer is referring to the Fine-Gray competing risk analysis using sub-distribution hazards models. This is a valid point brought up by the reviewer. However, we did perform a competing risk analysis since we analyzed the cause-specific risks, with the competing events of death and graft loss analyzed as censoring event. This is a valid method for taking into account competing risks (Lau B, Cole SR, Gange SJ. Competing risk regression models for epidemiologic data. Am J Epidemiol. 2009;170(2):244-256). In our study, we have a time-dependent, baseline immeasurable exposure (RBCT may occur at any point after transplant). Therefore, this is an “internal” covariate, where the change in exposure status relates to a biological change in the patient. When dealing with such variables, it has been suggested that the Fine-Gray sub-distribution hazard model may lead to results which are difficult to interpret, and thus not be the ideal method for accounting for competing risks (Austin PC, Latouche A, Fine JP. A review of the use of time-varying covariates in the Fine-Gray subdistribution hazard competing risk regression model. Stat Med. October 2019:sim.8399. doi:10.1002/sim.8399).**

3. Does TOH have a pre-operative policy for threshold Hb levels for transplant surgery to proceed?

**We do not have a strict Hb threshold for transplant to proceed. Generally, the decision to transfuse pre-transplant is decided amongst the surgeons, nephrology team and anesthetist. We have added this clarification in the Methods (see lines 146-149).**

4. In Table 1 the authors display the tacrolimus use (which I assume is time dependent with their period of study with most use after 2007) but what is the protocol for immunosuppression? Which anti-proliferatives are used and how are corticosteroids used at the center? I see this information is mentioned towards the end of the discussion but I think burden of immunosuppression is important to know.

**We have added this information in a supplemental file which summarizes our immunosuppression use in our kidney transplant recipients (see Supporting information, File S1).**

5. How would any blood transfusion and/or infection outside of TOH be captured?

**Any blood transfusion or infection occurring outside of our institution (TOH) would not have been captured in this study. We mention this limitation in our discussion (lines 396-400).**

6. What CMV prophylaxis is used at TOH? Is CMV and/or BK routinely checked post-transplant at certain time points?

**Indeed, this is good information to provide in the manuscript. We have added this in our Methods (lines 174-178)**

7. Do the authors think the association between incremental RBCT and bacterial infections is a surrogate for a kidney transplant patient with significant co-morbidity and/or complications that will render them susceptible to infections anyway? It may be hard to extract this data but any chance of knowing what the indication for giving RBCT was as this is likely to be a major confounder (and should be cited as such if this cannot be analysed).

**Indeed, this is a distinct possibility. We discussed the potential for such confounding, ways we tried to mitigate it and the possibility for unmeasured confounding (see Discussion, lines 359-365)**

**We unfortunately do not have information on the indication for giving RBCT. This would require extensive, meticulous chart review whereas we used our institution’s data warehouse to identify transfusion exposure. That being said, since the median (IQR) days to transfusion after transplant was 3 (2 to 6) days, we suspect the vast majority of these were given in the context of DGF. Our additional analyses stratifying by DGF status showed similar results to our original analysis. We have added this limitation as recommended in our Discussion (lines 400-404)**

8. The authors cite 2 meta-analyses on page 15 of the discussion with diametrically different outcomes - how were these two studies different to arrive at such different conclusions?

**The possible reason for those different findings were the inclusion of a large cardiac surgery trial in 2015 on restrictive vs liberal transfusion which showed no difference in infection risks. This trial came out between the 2 cited trials. We have added this information in our Discussion (lines 341-343).**
